# Suggestive

**Published:** 1878-06

**Authors:** 


### SUGGESTIVE.

FAST men, convivial men, men who love to "sport," generally have a short life, although it is apparently a merry one, for the bottle is an inseparable companion; in some form or other, brandy or wine is an indispensable item. Twelve years ago, a sporting party in one of its convivial moods buried a bottle of liquor, with the agreement that the survivor of them all, should repair to the spot, dig it up, and drink it to their memories. A few days ago, the last man performed the sad task. Only twelve years ago, and of all that merry company a single man remains! A temperate life, a life of religion expressing itself in daily good doing would have told a very different story.

